# Adherence evaluation of endocrine treatment in breast cancer: methodological aspects

**DOI:** 10.1186/1471-2407-12-474

**Published:** 2012-10-15

**Authors:** Anne S Oberguggenberger, Monika Sztankay, Beate Beer, Birthe Schubert, Verena Meraner, Herbert Oberacher, Georg Kemmler, Johannes Giesinger, Eva Gamper, Barbara Sperner-Unterweger, Christian Marth, Bernhard Holzner, Michael Hubalek

**Affiliations:** 1 Department of Psychiatry and Psychotherapy, Innsbruck Medical University, Anichstraße 35, 6020, Innsbruck, Austria; 2 Institute of Legal Medicine, Innsbruck Medical University, Anichstraße 35, 6020, Innsbruck, Austria; 3 Department of Obstetrics and Gynecology, Innsbruck Medical University, Anichstraße 35, 6020, Innsbruck, Austria

**Keywords:** Breast neoplasm, Endocrine therapy, Patient compliance, Method, Adherence

## Abstract

**Background:**

Current studies on adherence to endocrine therapy in breast cancer patients suffer from methodological limitations due to a lack of well-validated methods for assessing adherence. There is no gold standard for measuring adherence. The aim of our study was to compare four different approaches for evaluating adherence to anastrozole therapy for breast cancer with regard to concordance between methods.

**Methods:**

Outpatients with early breast cancer treated with anastrozole completed the multi-method assessment of adherence. We implemented a self-report scale (the Simplified Medication Adherence Questionnaire), physician- ratings, refill records and determination of anastrozole serum concentration.

**Results:**

Comparison of the four approaches using Spearman rank correlation revealed poor concordance across all methods reflecting weak correlations of 0.2-0.4. Considering this data incomparability across methods, we still observed high adherence rates of 78%-98% across measures.

**Conclusion:**

Our findings contribute to the growing body of knowledge on the impact that methodological aspects exert on the results of adherence measurement in breast cancer patients receiving endocrine treatment. Our findings suggest that the development and validation of instruments specific to patients receiving endocrine agents is imperative in order to arrive at a more accurate assessment and to subsequently obtain more precise estimates of adherence rates in this patient population.

## Background

Orally administered treatment with the new-generation aromatase inhibitors (AIs) plays an important role in the treatment of breast cancer resulting in substantial reductions in breast cancer recurrence [1,2]. In this regard it might be reasonable to assume breast cancer patients to be highly motivated and adherent to this treatment regime due to the seriousness of their disease, having “too much to lose” by not adhering [3]. Still, it has become apparent that, despite the great efficacy of AI treatment, non-adherence to adjuvant endocrine agents occurs frequently [4,5].

Evaluation of treatment adherence is, thus, a major issue in breast cancer care [4]. The assessment of long-term adherent behavior, however, is methodologically challenging. Studies have yielded inconclusive results indicating adherence rates between 20% and 100% across different phases of antineoplastic treatment [6,7]. This variability of non-adherence rates found in the literature has been suggested to be attributed to heterogeneous study designs as well as inconsistencies in methodological approaches. Among the latter the indirect methods of self-report, prescription refill and pharmacy records have been predominately used in studies on adherence to endocrine agents [3,4]. Direct methods which are supposed to reveal more objective results due to the assessment of medication consumption in an unmediated way have not been employed in respective studies. There is currently no Gold Standard of adherence measurement available [8].

Current evidence indicates that the patterns of findings on adherence might result more from the methods used to study them than from the underlying conceptual principles [9]. Highlighting the subsequent question as one of the most important ones in the field of adherence research, DiMatteo and Haskard [10] ask: *“How are findings [on adherence] influenced by the measurement strategies?”*

The type of measurement, whether direct or indirect [11], is an important determinant of the respective research finding [12]. DiMatteo and colleagues [13] noted in their review on patient adherence and treatment outcome that the adherence measurement variables of self-report, continuous measures and multi-method approaches moderated the adherence-outcome effects, which again reflects the dependence of results on the methodological approaches used. Moreover, both direct and indirect methods are prone to error – impacting on results. These methodological shortcomings seem to limit the appropriate evaluation of adherence rates for AI treatment, thereby hindering definitive conclusion and accounting for great data variability [3,11].

Accordingly, Cantrell [14] claimed the understanding of variations in adherence measurement to be an essential part of adherence research. Recognition and systematic assessment of the role of methodology seem to be of utmost importance to be able to draw conclusions on prevalence and correlates of (non-) adherence [10] as well as to evaluate adherence-interventions [15]. Direct comparison of the accuracy provided by various measures is, however, only possible if the methods are applied in the same patient population. In breast cancer patients receiving endocrine therapy, to the best of our knowledge, hardly any studies have compared various adherence measures in relation to their concordance [3,16].

### Aims

In this study, we aimed at comparing four adherence measurement methods, all of which were defined by the WHO as state-of-the-art measurement of adherence [8], for their concordance in breast cancer patients undergoing anastrozole treatment. We compared self-report, physician rating, determination of plasma concentration and prescription refill.

In detail, we have addressed the following research questions:

● Do different adherence measures reveal comparable results?

● To what extent do early breast cancer patients adhere to AI treatment?

## Methods

This study is part of a larger project referred to as "Patient-reported outcomes in breast cancer patients undergoing endocrine therapy: an observational study of adherence (PRO-BETh)" at the Medical University Innsbruck. The aim of PRO-BETh is to perform a multi-method evaluation of adherence to endocrine treatment in pre- and post-menopausal breast cancer patients. Physical symptoms and psychosocial burden are assessed using patient-reported outcome (PRO) measures and analyzed in relation to their impact on adherence. In addition, plasma concentrations of endocrine treatment and pharmacogenetic aspects were investigated. For the whole PRO-Beth study a total of 563 patients were approached comprising pre- and postmenopausal patients receiving any kind of endocrine treatment. 70 patients declined to participate resulting in an overall participation rate of 87.6%. Data on physical symptoms and psychosocial burden (PRO assessment) in the postmenopausal patient group receiving any kind of aromatase inhibitor treatment have been published elsewhere [17]. Analyses on symptom burden in premenopausal patients receiving tamoxifen as well as on pharmacogenetic aspects have not yet been completed. For the analysis presented herein, we only included patients receiving anastrazole (not adjusted for menopausal state) to provide group homogeneity.

Approval was obtained from the Ethics Committee of Medical University Innsbruck.

### Sample

Breast cancer outpatients treated at the Department of Gynecology and Obstetrics (Innsbruck Medical University) between June 2009 and February 2011 were considered for enrolment. Patients were eligible for this substudy of the PRO-BETh if they

– had a diagnosis of non-metastatic breast cancer,

– were undergoing adjuvant endocrine therapy with anastrazole (>0.5 months after their primary treatment)

– had no prior treatment with any endocrine agent or any overt cognitive impairment

– were aged between 18 and 85 and

– were fluent in German and

– provided written informed consent.

Eligible patients were identified by searching the department's medical records.

### Procedure

Patients were approached at one of their routine three-month follow-up appointments at the Department of Gynecology and Obstetrics, Medical University Innsbruck. While waiting for their appointment, patients completed the PRO-assessment of adherence (self-report questionnaire) and Quality of Life (QOL). Further details and results of the PRO-assessment are described elsewhere [17]. Blood samples were collected after the completion of the questionnaires as part of routine blood collection and subsequently sent to the Department of Forensic Medicine (Medical University Innsbruck) for analysis. Then, the routine medical check-up was conducted after which the physicians completed their adherence rating. All adherence data were collected cross-sectionally with exception of those derived from the refill records. The latter were collected retrospectively for each patient (details are provided in the chapter assessment instruments).

Clinical and sociodemographic variables were taken from the clinical records. Refill records were obtained from the insurance companies at the end of the assessment period.

Adherence was defined as a function of the instrument applied.

### Assessment methods

A main focus of this study was the comparison of commonly used assessment methods for adherence. These include self-report measures (SMAQ), proxy ratings by the physician, refill records.

As a further measure for adherence we determined AI-plasma concentrations. To the best of our knowledge, this is the first study assessing AI-plasma concentrations for the evaluation of AI adherence.

### Self-report questionnaire (SMAQ)

For patient self-report, we decided to administer the *Simplified Medication Adherence Questionnaire* (SMAQ) originally validated in an HIV population [18], since there is no questionnaire available that specifically assesses treatment adherence to endocrine treatment for early breast cancer. The SMAQ is a six-item, short self-report questionnaire measuring the overall trend of chronically ill patients' medication adherence and the accuracy of medication intake (e.g. number of missed doses). It is composed of the revised Morisky Scale (3 items in the original version) [19] and three additional items which were supplemented by the developers of the SMAQ. The following six questions compose the SMAQ: 1. Do you ever forget to take you medicine? (response format: yes-no), 2. Are you careless at times about taking your medicine? (response format: yes-no), 3. Sometimes if you feel worse, do you stop taking your medicines? (response format: yes-no), 4. Think back to last week. How often did you not take your medicine? (5 point likert scale: “never” to more than “10 times”), 5. Did you fail to take any of your medicine over the past weekend? (response format: yes-no), 6. Over the past three months, on how many days did you not take any medicine at all? (resonse format: less than 2 times-more than two times). According to the SMAQ a patient is considered non-adherent in case of a positive response (“yes”) to any of the questions 1–3 and 5 or/and more than two doses missed over the past week (item 4) or/and more than 2 days of non-medication intake during the past 3 months (item 6) [18].

The SMAQ was translated into German following a forward-backward translation process and adapted to the requirements of this study by developing a scoring system to replace the rigorous, dichotomous outcome (adherent-non adherent). We developed a sum score across all questions by dichotomizing all items (score 1 = non adherent, score 2 = adherent). The scores were then added to the final score with a possible range between 6 and 12. The dichotomization was done in the following: For item 6 the answer “less than two times” was scored with 2 (adherent); for item 4 the answer “never” was scored with 2 (adherent) while the other categories were classified as non-adherent (score 1). Item 1–3 and 5 stayed with the original dichotomous response (no = score 2 = adherent; yes = score 1 = non-adherent). Only patients with the highest possible sum score (i.e. 12) were classified as adherent. This corresponds to the 90% percentile which was chosen following recommendations in the literature [20]. With a moderate Cronbach’s Alpha of 0.67, the questionnaire proved to be moderately reliable in this specific patient population. Patients completed the questionnaire themselves.

### Physician rating

For each patient, the clinical expert rating was performed by the treating gynecologist/oncologist after the patient’s three-month routine check-up via a dichotomous classification yes (adherent) – no (non-adherent) (same day of the other assessments). The experienced physician classified patients as adherent or non-adherent based on his or her clinical impression. Medical expert ratings are an established method for assessing adherence [8], in particular in daily clinical practice.

### Health insurance data on filled prescriptions (pharmacy refill)

The health insurance company provided records on filled prescriptions for AIs for each patient. Patients were supposed to collect one pill package per month (contains 30 pills per package) at the pharmacy. E.g. the should-be-value for 6 months is 6. The medication-possession ratio (MPR) determined the number of prescriptions the study patients actually collected at the pharmacy (and that were then submitted to the health insurer) in relation to the should-be value for six months previous to the assessment as well as for the period from the first prescription to the assessment time-point. Patients who refilled their prescription more than 90% of the time were considered adherent and those who refilled it less than 90% of the time non-adherent. The selection of this cut-off is in accordance with recommendations in the adherence literature [20]. These Time gaps spent without medication were self-evident.

### Determination of AI plasma concentrations

For the determination of anastrozole plasma concentrations, a liquid chromatography tandem mass spectrometry method was developed and fully validated according to the guidelines for clinical and forensic toxicology. Methodological procedures have been described elsewhere [21]. Anastrozole plasma concentrations below the limit of quantification were defined as indicative for non-adherence.

### Statistical analysis

Sample characteristics are given as frequencies, means, standard deviations and ranges. Adherence rates are presented as percentage with confidence intervals for each assessment method in detail. Confidence intervals are calculated using the adjusted Wald method. Differences between adherence rates derived from the different methodological measurement approaches were investigated using McNemar test.

To investigate the employed methods for adherence measurement with regard to consistency we used the Spearman rank correlation. For these analyses, we also adjusted for age and treatment duration using partial correlation.

We additionally determined the impact of the differing temporal reference-frames of the methods applied on methodological comparability. For this purpose, we varied the time-frames for the observation period of medication intake derived from the insurance as follows: previous month, previous 3 months, previous 4 months and previous 6 months to the assessment time-point. Additionally, we extracted the single questions of the SMAQ referring to the time-frames of intake of last weekend, the previous week and the previous 3 months; we separately analyzed the association of these single items as well as the different time-frames of the insurance data with the other methods (physician ratings, plasma concentrations) by means of Spearman rank correlation.

## Results

### Patient characteristics

From June 2009 to May 2010, 276 breast cancer patients receiving anastrazole were identified as eligible for inclusion in this analysis. A total of 242 patients provided written informed consent, 34 (12.3%) declined to participate. Main reasons for non-participation were organizational and logistic problems. There were no significant differences between patients who participated and those who declined with regard to clinical and sociodemographic variables.

Patients varied in age between 40 and 84 years, with a median age of 65.0 (SD 8.3) years. Median treatment duration was 26.7 months (SD 19.1, range 0.7-89.5) on average. About 8% of patients had been undergoing anastrazole treatment for >5 years due to their participation in a trial investigating the efficacy of extended treatment duration. The most frequent histopathologic cancer type in this study sample was invasive carcinoma (93.6%), the most frequent grade was grade II carcinoma (72.7%). For further details, see Table 1.

**Table 1:** Clinical and sociodemographic data (N = 242)

		**Frequency (%)**
*Age*	Median (SD)	65.00 y (8.3y)
	Range	40-84y
*Marital status*	Single	18 (8.4%)
	Partnership, marriage	135 (63.1%)
	Divorced, separated	28 (13.1%)
	Widowed	33 (15.4%)
*Employment status*	Full-time	16 (7.5%)
	Part-time	16 (7.5%)
	Unemployed	5 (2.1%)
	Homemaker	31 (14.5%)
	Retired	142 (66.4%)
	Other	4 (1.6%)
*Histological classification*	Invasive	226 (93.4%)
	In situ	15 (6.2%)
	Missing	1 (0.4%)
*Grading*	Grade I	35 (14.5%)
	Grade II	176 (72.7%)
	Grade III	10 (4.1%)
	Unknown	21 (8.7%)
*Duration of adjuvant endocrine therapy (months)*	Median (SD)	26.7 (19.1) mo.
	Range	0.7- 89.5 mo.
*Primary surgical treatment*	Breast-conserving procedure	155 (65.7%)
	Mastectomy	81 (34.3%)
	Reconstruction	43 (18.2%)
*Chemotherapy*		41 (16.9%)
*Radiotherapy*		169 (71.3%)

**Figure 1:**
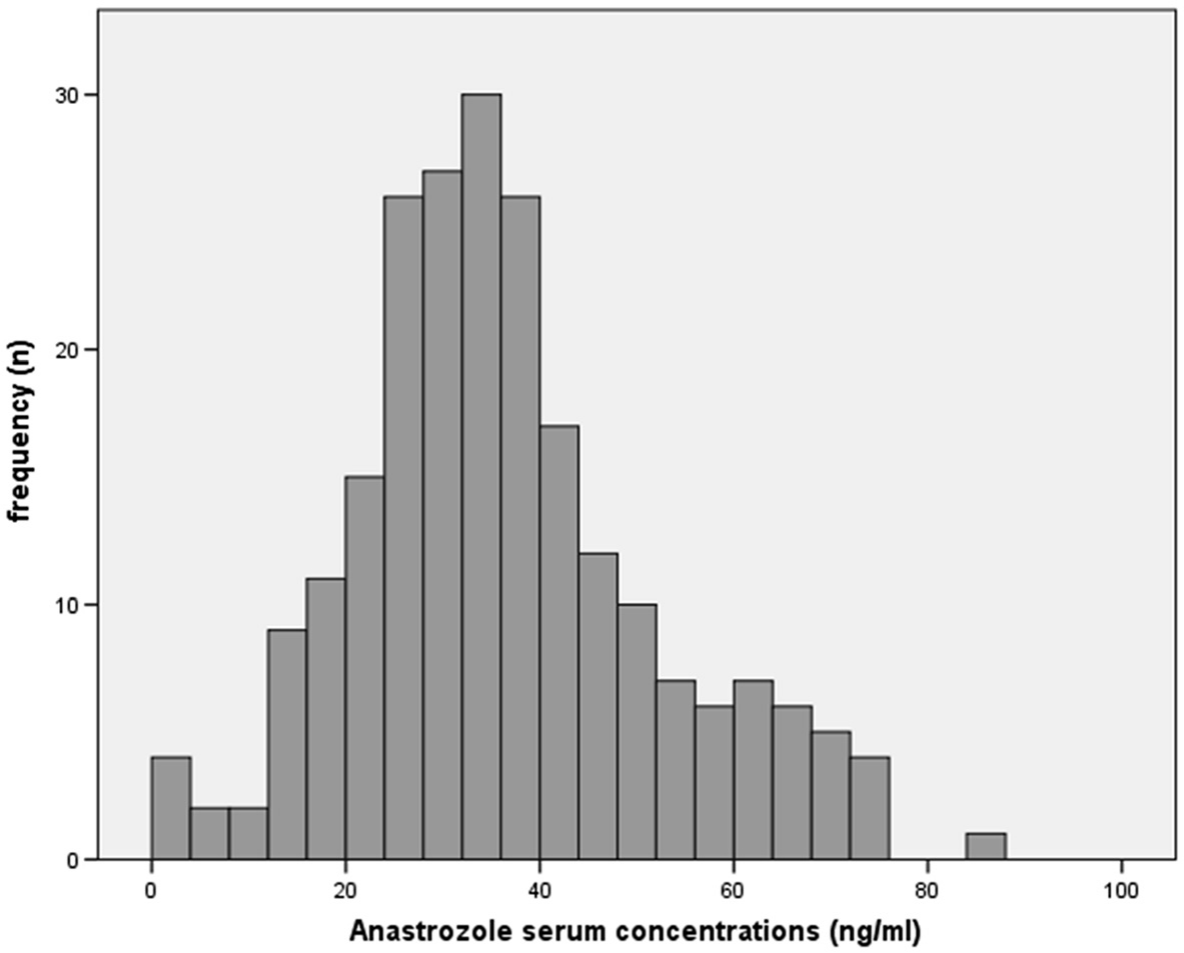
Distribution of anastrazole serum concentrations (presented in ng/ml).

The determined AI plasma concentrations ranged between 5.4 and 90.7 ng/ml. The plasma samples of four patients showed concentrations below the quantification limit. Please find details on the distribution of anastrazole serum concentrations in Figure 1. Further details have been presented elsewhere [21].

### Comparison of methods for adherence measurement

The comparison of self-reported adherence, physician-rated adherence, prescription refill and AI plasma concentration revealed moderate concordance between methods. Overall, significant (*p* < 0.05) correlations ranging from r = 0.189 to r = 0.369 were found across all methods. Highest correlations were seen between physician ratings and self-reports, namely r = 0.369 (*p* < 0.001), lowest between prescription refill and self-report (r = 0.189, p = 0.050). Details are listed on Table 2.

**Table 2:** Correlation of methods for adherence measurement

	**Self-report (n = 186)**		**Physician rating (n=211)**		**Prescription refill (n=121)**		**Plasma concentration (n=223)**	
	**r**	***p***	**r**	***p***	**r**	***p***	**r**	***p***
*Self-report*	1	-	0.369	<0.001*	0.189	0.050*	0.223	0.002*
*Physician rating*			1	-	0.243	0.006*	0.265	<0.001*
*Prescription refill*					1	-	0.251	0.005*
*Plasma concentration*							1	-

*indicates significant correlation (*p*<0.05).

The adjustment for treatment duration and age by means of partial correlation had no relevant effect on results regarding the comparison of methods (results not shown).

### Impact of different time-frames/ temporal reference-frames on concordance between methods

Analyses of the impact of the differing time-frames of the methods applied for adherence measurement revealed the following results: The shorter the time-intervals for refilled prescriptions were defined, the smaller was the correlation with the other methodological approaches (see Table 3). The SMAQ-question referring to a medication intake over the previous 3 months was not significantly associated with the 3-month time-interval of prescription refill (r = 0.120, p = 0.183) but significantly with the 6-month interval (r = 0.197, p = 0.038). Additionally, the 6-month time-frame for medication intake was not associated with the SMAQ question for medication intake at the previous weekend (r = −0.13, p = 0.889).

**Table 3:** Impact of differing time-frames on correlations between methods

**Prescription refill**	**Physician rating**		**Plasma concentration**		**Self-report**	
	**r**	**p**	**r**	**p**	**r**	**p**
*Prescription refill – previous month*	0.090	0.349	−0.071	0.470	0.112	0.276
*Prescription refill – 3months*	0.105	0.226	0.050	0.573	0.115	0.218
*Prescription refill – 4months*	0.194	0.021*	0.175	0.040*	0.167	0.069
*Prescription refill – 6months*	0.243	0.006*	0.251	0.005*	0.189	0.050^#^

*indicates significant correlation: *p*<0.05, ^#^ indicates significant correlation: *p*=0.050.

These results suggest a 6-month time-frame for the method of prescription refill, to better capture adherence behavior compared to shorter time-frames.

### Evaluation of adherence to AI

Overall, high adherence rates of 77.8%-98.2% were observed across all assessment methods (see Table 4). Physician ratings revealed highest adherence rates (98.2%), and overall prescription refill the lowest adherence rates (78%). We further determined differences of adherence rates between methods using the McNemar test and observed significantly higher adherence rates indicated by plasma concentration compared to the other methodological approaches. Also adherence rates derived from physicians’ ratings were significantly higher than self-reported adherence and adherence indicated by prescription refill. Further details are summarized Table 4.

**Table 4:** Overall adherence rates across methods

**Assessment Method**	**Number of patients (%)**	
	**Adherent**	**CL 95%**
Plasma concentration ^a^	98.2%	95-99%
Physician rating	92.1%*	88-95%
Prescription refill (6months)^b^	85.3%*	78-90%
Self-rating	82.6%*^+^	77-87%
Overall prescription refill ^b^	77.8%*^+^	70-83%

^a^ plasma concentrations below the quantification limit indicate non-adherence (see Methods section).

^b^ an MPR of >90% was classified as adherent.

* significant lower adherence rate than adherence rate indicated by plasma concentration.

^+^ significant lower adherence rate than adherence rate indicated by physician-rating.

## Discussion

In recent years, the use of oral anticancer treatment, particularly preventive treatments such as AI therapy, has been expanding and it is likely to further increase in the future [22]. Patient adherence is essential for the success of disease management [23], but should not be taken for granted, as growing evidence on non-adherence indicates. Drawing conclusions on actual adherence rates in breast cancer patients is limited by considerable discordance in results due to methodological inconsistency across studies and by the various measurement methods employed.

Herein, we report on a comparison of four methodological approaches for adherence assessment in patients receiving AI treatment in order to investigate consistency of results determined by self-report, physician rating, refill records and the measurement of substance plasma concentrations. To the best of our knowledge, this is the first study directly comparing these four measurement approaches in this patient population.

The methods implemented in our study revealed adherence rates between 78% and 98%, reflecting well-known inconsistencies for estimations of adherence to endocrine agents. These numbers are in the higher bracket of the 20%-100% range reported in the literature [6,7]. Chlebowski and colleagues (2006) quote adherence rates of 72%-77% across adjuvant clinical breast cancer trials and of 54%-80% in breast cancer prevention trials, regardless of method or type of endocrine agent. However, very recent studies implementing single methods of adherence assessment report highly contradictory results [24,25]. This might be attributed to the heterogeneity of study designs. Therefore, because of the remarkable heterogeneity of reported adherence rates and of methods of determination used, a comparison of the different methods appears to be of particular relevamce.

Our study results suggest an at most modest concordance between adherence measures tested in a sample of breast cancer patients receiving endocrine treatment. Given correlations of 0.2-0.4, the comparability of results using these study methods is rather limited.

This observation is consistent with the literature on adherence in chronic disease. In a study by Dunbar-Jacobs and colleagues [26] the comparison of self-report (7-day recall), MEMS (Medication Event Monitoring System) and pill count to assess adherence to lipid-lowering medications revealed a lack of correlation between measures despite sample homogeneity (regarding observation period and treatment). Additionally, the association between self-report and MEMS in a sample of rheumatoid arthritis patients approached zero [9].

Evidence on the subject of comparability of methodological approaches for measurement of adherence to AIs is scarce. Ziller and colleagues [16,27] reported a significant gap between self-reports and prescription refills for adherence to tamoxifen and anastrozole. They found non-adherence rates of 20%-31% on the basis of prescription refill records and perfect self-reported adherence (100%). In an earlier study by Waterhouse and colleagues [3] in 24 patients receiving tamoxifen, 98% were classified as adherent using self-reports and 92% using pill counts, but only 69% by means of MEMS. The self-report questionnaire and pill count taken together identified about 17% of patients with poor adherence, whereas MEMS found that 75% of the cohort was less than 80% adherent to the tamoxifen regimen.

Our findings suggest a strong dependence of the estimated adherence rates on the specific measurement method used. As assumed in previous studies [3,16] methodological shortcomings assigned to each measurement approach may serve as an explanation for these discrepancies. The following limitations need to be considered when interpreting our findings:

Two major attributes of the determination of *substance plasma concentrations* are its high level of accuracy and objectiveness [28,29]. Since concentrations of one single anastrozole dose can be detected several days after cessation (mean anastrozole elimination half-life 45.4 – 50 h [30]) the mere verification of the substance is not indicative of regular medication intake. On the other hand, inter-patient variations in AI elimination can cause highly variable plasma concentrations despite regular drug intake. Farmer et al. [31] suggested that this method simply shows whether the patient recently took a dose of the drug, but does not quantify the manner in which the patient took the drug or any fluctuation in medication intake. Concurrently, interindividual differences in drug pharmacokinetics challenge an appropriate definition of a threshold for plasma concentrations indicating (non-) adherence. Urquhart [2] claims biological variability in drug response to be the main obstacle to using biological markers for adherence.

Moreover, a lack of evidence on those plasma level relationship limits the interpretation of individual values. Beer and colleagues [21] suggested that those patients with values below or close to the 10^th^ percentile of the study population are likely not adhering to the regime prescribed, regardless of time since intake. Nonetheless, they claim concentrations at the lower end of the distribution to be indicative, but not confirmative, of non-adherence and recommend repeated assessments as a more qualified strategy for assessing adherence. Therefore, classifying only those patients with plasma concentrations below the quantification limit as non-adherent we may have underestimated non-adherence behavior in this patient population. Moreover, this approach does not consider interindividual differences for metabolization. In order to be able to use the determination of AI plasma concentrations to evaluate adherence, clear thresholds for adequate plasma concentrations are needed. On the other hand by using such a conservative approach, we clearly reduced the site of overestimating adherence.

Results based on *prescription refills* strongly depend on the threshold for the medication-possession ratio defined as indicating adherence. The determination of an adequate medication-possession ratio is recommended inconsistently in the available literature, namely between 80% and 95%. The level of medication intake required to achieve a therapeutic efficacy of endocrine agents is still unclear and challenges the definition of this threshold. In contrast to Ziller and colleagues [16] and Waterhouse and colleagues [3], we defined a value of ≥ 90% as satisfactory following Dunbar-Jacob and Sereika [20], who claimed the majority of patients to adhere above the 90% level. This percentage appeared to be persistent across measures and over time and was thus regarded as adequate for this study. However, the key obstacle presented by this measure is the underlying premise that refill equals medication intake. This premise is invalid if patients refill their medication, particularly in the case of low out-of-pocket costs, but are reluctant regarding intake. Moreover, patients who never filled any prescription or developed metastatic disease are lost to this approach.

Lacking a validated *self-report questionnaire* specific for breast cancer patients receiving AIs we used the SMAQ, which considers the impact of iatrogenic harm. After adaptation for use in our patient sample, a German version of the questionnaire was deemed appropriate for patients receiving any type of endocrine treatment. Yet, we are aware of the shortcoming of lacking validation. In this context, the inconsistent use of a great variety of self-report measures further limits data comparability [31-34].

*Proxy-rated adherence* by the treating physician can be susceptible to bias in terms of overestimation. Treating clinicians seem to be at risk for inaccurately estimating adherence, either because they are unaware of their patient‘s poor intake behavior or simply underestimate it [35-37]. The fact that the second highest adherence rates in the present study were derived from the proxy ratings partly support this assumption.

Besides the limitations distinctive to each measurement method, there are some more general methodological aspects of this study which might contribute to the observed difference between methods. First, the selection of non-adherence cut-offs is somewhat arbitrary for all methods which impacts on prevalence rates. Nonetheless, the subject of defining satisfactory cut-off levels is a common problem in adherence research and has been heterogeneously discussed in the literature. Second, differing time-frames of adherence measurement methods could have impacted on the comparability of the approaches. However, we considered this subject by varying time-frames and could illustrate differing time-frames to have little effect on methodological comparability. Finally, acknowledging the shortcomings of a cross-sectional design for the investigation of an issue that might be influenced by time (of medication intake), our results elucidate the complexity of adherence measurement.

## Conclusion

In conclusion, this study helps elucidating some of the underlying reasons for discrepancies of reported adherence data for endocrine agents. Our findings show that the impact of methodology used is of considerable importance when investigating adherence. In order to arrive at a more accurate assessment and to subsequently obtain more precise estimates of adherence rates in this patient population, it is mandatory to develop and validate instruments specific to patients receiving endocrine agents for breast cancer treatment. In addition, the determination of AI-serum concentrations at regular intervals may provide a meaningful measure for patients’ adherence.

## Competing interests

'The authors declare that they have no competing interests'.

## Authors’ contribution

AO participated in the design of the study, performed the statistical analysis drafted the manuscript. MS participated in the study coordination, conducted the data collection and helped to draft the manuscript. VM participated in the design of the study, in study coordinations and conceived of the study. JG conceived of the study. GK participated in the design of the study and contributed to the statistical analysis. MH participated in the design of the study and helped to draft the manuscript. BH conceived of the study, and participated in its design. BS-U conceived of the study, and participated in its design. EG participated in study coordination, CM conceived of the study. BB conducted the analytical procedures (determination of AI plasma concentrations) and participated in the design of the study. HO conducted the analytical procedures (determination of AI plasma concentrations) and participated in the design of the study. BS conducted the analytical procedures (determination of AI plasma concentrations). All authors read and approved the final manuscript.

## Pre-publication history

The pre-publication history for this paper can be accessed here:

http://www.biomedcentral.com/1471-2407/12/474/prepub

## References

[B1] Cuzick J Sestak I Baum M Buzdar A Howell A Dowsett M Forbes JF investigators AL Effect of anastrozole and tamoxifen as adjuvant treatment for early-stage breast cancer: 10-year analysis of the ATAC trial Lancet Oncol 2010 11 12 1135 1141 10.1016/S1470-2045(10)70257-6 21087898

[B2] Urquhart J Ockene LBI Biological measures Compliance in Healthcare and Research. edn 2001 Futura Publishing Company, New York 105 117

[B3] Waterhouse DM Calzone KA Mele C Brenner DE Adherence to oral tamoxifen: a comparison of patient self-report, pill counts, and microelectronic monitoring J Clin Oncol 1993 11 6 1189 1197 8501505 10.1200/JCO.1993.11.6.1189

[B4] Chlebowski RT Geller ML Adherence to endocrine therapy for breast cancer Oncology 2006 71 1–2 1 9 17344666 10.1159/000100444

[B5] Hershman DL Shao T Kushi LH Buono D Tsai WY Fehrenbacher L Kwan M Gomez SL Neugut AI Early discontinuation and non-adherence to adjuvant hormonal therapy are associated with increased mortality in women with breast cancer Breast Cancer Res Treat 2011 126 2 529 537 10.1007/s10549-010-1132-4 20803066 PMC3462663

[B6] Partridge AH Non-adherence to endocrine therapy for breast cancer Ann Oncol 2006 17 2 183 184 16428243 10.1093/annonc/mdj141

[B7] Partridge AH Avorn J Wang PS Winer EP Adherence to therapy with oral antineoplastic agents J Natl Cancer Inst 2002 94 9 652 61 10.1093/jnci/94.9.652 11983753

[B8] WHO Adherence to long-Â­term therapies, evidence for action 2003 Marketing and Dissemination, Geneva

[B9] Dunbar-Jacob J Mortimer-Stephens MK Treatment adherence in chronic disease J Clin Epidemiol 2001 54 Suppl 1 S57 S60 11750211 10.1016/s0895-4356(01)00457-7

[B10] DiMatteo MR Haskard KB Further challenges in adherence research: measurements, methodologies, and mental health care Med Care 2006 44 4 297 299 10.1097/01.mlr.0000214527.98190.2a 16565628

[B11] Osterberg L Blaschke T Adherence to medication N Engl J Med 2005 353 5 487 497 10.1056/NEJMra050100 16079372

[B12] DiMatteo MR Evidence-based strategies to foster adherence and improve patient outcomes JAAPA 2004 17 11 18 21 15575518

[B13] DiMatteo MR Giordani PJ Lepper HS Croghan TW Patient adherence and medical treatment outcomes: a meta-analysis Med Care 2002 40 9 794 811 10.1097/00005650-200209000-00009 12218770

[B14] Cantrell CR Eaddy MT Shah MB Regan TS Sokol MC Methods for evaluating patient adherence to antidepressant therapy: a real-world comparison of adherence and economic outcomes Med Care 2006 44 4 300 303 10.1097/01.mlr.0000204287.82701.9b 16565629

[B15] Doggrell SA Adherence to medicines in the older-aged with chronic conditions: does intervention by an allied health professional help? Drugs Aging 2010 27 3 239 254 10.2165/11532870-000000000-00000 20210369

[B16] Ziller V Kalder M Albert US Holzhauer W Ziller M Wagner U Hadji P Adherence to adjuvant endocrine therapy in postmenopausal women with breast cancer Ann Oncol 2009 20 3 431 436 19150950 10.1093/annonc/mdn646

[B17] Oberguggenberger A Hubalek M Sztankay M Meraner V Beer B Oberacher H Giesinger J Kemmler G Egle D Gamper EM Is the toxicity of adjuvant aromatase inhibitor therapy underestimated? Complementary information from patient-reported outcomes (PROs) Breast Cancer Res Treat 2011 128 2 553 561 10.1007/s10549-011-1378-5 21311968

[B18] Knobel H Alonso J Casado JL Collazos J González J Ruiz I Kindelan JM Carmona A Juega J Ocampo A Validation of a simplified medication adherence questionnaire in a large cohort of HIV-infected patients: the GEEMA Study AIDS 2002 16 4 605 613 10.1097/00002030-200203080-00012 11873004

[B19] Morisky DE Green LW Levine DM Concurrent and predictive validity of a self-reported measure of medication adherence Med Care 1986 24 1 67 74 10.1097/00005650-198601000-00007 3945130

[B20] DunbarÂ­Jacob J Sereika S Ockene L, Burke I Conceptual and methodological problems Compliance in Healthcare and Research 2001 Futura Publishing Company, New York 93 106

[B21] Beer B Schubert B Oberguggenberger A Meraner V Hubalek M Oberacher H Development and validation of a liquid chromatography-tandem mass spectrometry method for the simultaneous quantification of tamoxifen, anastrozole, and letrozole in human plasma and its application to a clinical study Anal Bioanal Chem 2010 398 4 1791 1800 10.1007/s00216-010-4075-z 20730580

[B22] DiMatteo MR Haskard KB Williams SL Health beliefs, disease severity, and patient adherence: a meta-analysis Med Care 2007 45 6 521 528 10.1097/MLR.0b013e318032937e 17515779

[B23] Hershman DL Kushi LH Shao T Buono D Kershenbaum A Tsai WY Fehrenbacher L Lin Gomez S, Miles S, Neugut AI: Early discontinuation and nonadherence to adjuvant hormonal therapy in a cohort of 8,769 early-stage breast cancer patients J Clin Oncol 2010 28 27 4120 4128 10.1200/JCO.2009.25.9655 20585090 PMC2953970

[B24] Huiart L Dell'Aniello S Suissa S Use of tamoxifen and aromatase inhibitors in a large population-based cohort of women with breast cancer Br J Cancer 2011 104 10 1558 1563 10.1038/bjc.2011.140 21522148 PMC3101914

[B25] Dunbar-Â­‐Jacob J Comparability of self-Â­‐report, pill count and electronically monitored adherence data Controlled Clinical Trials 1996 12 Supplement 2

[B26] Ziller V Wetzel K Kyvernitakis I Seker-Pektas B Hadji P Adherence and persistence in patients with postmenopausal osteoporosis treated with raloxifene Climacteric 2011 14 2 228 235 10.3109/13697137.2010.514628 20964548

[B27] Larson ME Richards TM Quantification of a methadone metabolite (EDDP) in urine: assessment of compliance Clin Med Res 2009 7 4 134 141 10.3121/cmr.2009.859 19920164 PMC2801695

[B28] van Rossum AM Bergshoeff AS Fraaij PL Hugen PW Hartwig NG Geelen SP Wolfs TF Weemaes CM De Groot R Burger DM Therapeutic drug monitoring of indinavir and nelfinavir to assess adherence to therapy in human immunodeficiency virus-infected children Pediatr Infect Dis J 2002 21 8 743 747 10.1097/00006454-200208000-00009 12192162

[B29] Yates RA Dowsett M Fisher GV Selen A Wyld PJ Arimidex (ZD1033): a selective, potent inhibitor of aromatase in postmenopausal female volunteers Br J Cancer 1996 73 4 543 548 10.1038/bjc.1996.94 8595172 PMC2074469

[B30] Farmer KC Methods for measuring and monitoring medication regimen adherence in clinical trials and clinical practice Clin Ther 1999 21 6 1074 1090 discussion 1073 10.1016/S0149-2918(99)80026-5 10440628

[B31] Fink AK Gurwitz J Rakowski W Guadagnoli E Silliman RA Patient beliefs and tamoxifen discontinuance in older women with estrogen receptor–positive breast cancer J Clin Oncol 2004 22 16 3309 3315 10.1200/JCO.2004.11.064 15310774

[B32] Demissie S Silliman RA Lash TL Adjuvant tamoxifen: predictors of use, side effects, and discontinuation in older women J Clin Oncol 2001 19 2 322 328 11208822 10.1200/JCO.2001.19.2.322

[B33] Kahn KL Schneider EC Malin JL Adams JL Epstein AM Patient centered experiences in breast cancer: predicting long-term adherence to tamoxifen use Med Care 2007 45 5 431 439 10.1097/01.mlr.0000257193.10760.7f 17446829

[B34] Murthy V Bharia G Sarin R Tamoxifen non-compliance: does it matter? Lancet Oncol 2002 3 11 654 10.1016/S1470-2045(02)00895-1 12424066

[B35] Miller LG Hays RD Measuring adherence to antiretroviral medications in clinical trials HIV Clin Trials 2000 1 1 36 46 10.1310/ENXW-95PB-5NGW-1F40 11590488

[B36] Clayton CD Veach J Macfadden W Haskins J Docherty JP Lindenmayer JP Assessment of clinician awareness of nonadherence using a new structured rating scale J Psychiatr Pract 2010 16 3 164 169 10.1097/01.pra.0000375712.85454.c6 20485104

[B37] Hugen PW Langebeek N Burger DM Zomer B van Leusen R Schuurman R Koopmans PP Hekster YA Assessment of adherence to HIV protease inhibitors: comparison and combination of various methods, including MEMS (electronic monitoring), patient and nurse report, and therapeutic drug monitoring J Acquir Immune Defic Syndr 2002 30 3 324 334 12131570 10.1097/00126334-200207010-00009

